# Health Tract, No. 52

**Published:** 1861-11

**Authors:** 


### HEALTH TRACT, No. 52.

*From Hall's Journal of Health*, 42 *Irving Place.* $1 *a year.*

### CATARRH

Is a name given by the Greeks to ailments which throw off fluids in unnatural quantities; it means a "flowing from." These catarrhs are always originated by a cold taken in some way; and upon whatever part of the system the cold "falls," it is called a "catarrh" of that part. Hence, "catarrh of the head" when the eyes water a great deal; "nasal" catarrh when the "nose runs;" "catarrh of the chest" when a cold settles on the lungs and a large expectoration follows. Some persons who have "weak bowels" always have diarrhea; thin, watery, light-colored passages, or catarrh of the bowels, when a cold is taken.

The action of a catarrh is curative, and should be let alone, for it is nature's effort to carry off the disease; to wash it away, as it were. If nature were only left to herself in these cases, an incredible amount of suffering would be prevented, especially if nothing were eaten until relieved but bread and water; and if two or three hours in the forenoon and afternoon were spent in the open air, in bodily activities sufficient to promote and keep up a very gentle perspiration. But when there is a cough, or a troublesome running at the nose, or a watering of the eyes, with a fullness about the head and all over the body, indicating that a general cold has been taken, there is almost a mania for "taking something;" or, if the person has some medical knowledge, and even a small amount of common-sense, leading him to wait on nature, while he endeavors to aid her as just indicated, every second person he meets, exclaims, "Why don't you do something for it?" and he is brave indeed who resists steadfastly to the end.

A lady had a troublesome itching and running at the nose, and being advised to snuff up cold water freely, she did so and was "cured" in a day; but in twenty-four hours she nearly died of asthma; for, although the "flowing" from the nose was checked, the disease fell upon the lungs; nature would have vent some where.

In the diarrheas of children, summer complaints, etc., which so often arise from colds settling on the bowels, paregoric is given, and "soothing syrups," (in ALL cases made of molasses and laudanum, *never* made without sugar and opium.) The great effort of ignorance is to "stop the diarrhea." This is done; the parents are charmed, write out a certificate in great gratitude; this is published in the morning papers of the same week, as also in another column the death of the "cured" child of "convulsions" or "water on the brain."

The cough of consumption, and the large amount of glairy or multi-colored "matter" discharged from the lungs in bronchitis, are the curative "flowings," catarrhs of nature, and the checking of them by cough-drops, lozenges, troches, syrups, snuffs, etc., *always*, ALWAYS, ALWAYS makes death more certain, more speedy, and more dreadful. In all catarrhs, in all flowings, keep the bowels free; keep up a very general perspiration, and eat but very little for forty-eight hours, and if not better, send for a respectable physician.

